# Supplementary material for: Functional connectivity is linked to symbolic BOLD patterns: Replication, extension, and clinical application of the human “Complexome”
Source: Netw Neurosci. 2026 Aug 25;10(3):761–86. doi: 10.1162/NETN.a.572 (PMC13569379; doi:10.1162/NETN.a.572)
Supplement: Supplementary file 1 [file netn-10-3-761-s001.pdf]

SUPPLEMENTARY MATERIAL
for

Functional connectivity is linked to symbolic BOLD patterns: replication, extension, and clinical application of the human 'complexome'

Amy Romanello[1,2], Nina von Schwanenflug[1,2], Michelle Franka[1,2], Friedemann Paul[3,4,5], Harald Prüss[1,6], Stephan Krohn[1,2†], Carsten Finke[1,2†*]

[1] Department of Neurology and Experimental Neurology, Charité-Universitätsmedizin Berlin, corporate member of Freie Universität Berlin, Humboldt-Universität Berlin, Berlin, Germany.

[2] Berlin School of Mind and Brain, Humboldt-Universität zu Berlin, Berlin, Germany.

[3] Max Delbrück Center for Molecular Medicine in the Helmholtz Association, Berlin, Germany.

[4] Experimental and Clinical Research Center, a cooperation between the Max Delbrück Center for Molecular Medicine in the Helmholtz Association and Charité—Universitätsmedizin Berlin, Berlin, Germany.

[5] NeuroCure Clinical Research Center, Charité—Universitätsmedizin Berlin, corporate member of Freie Universität Berlin, Humboldt-Universität zu Berlin, and Berlin Institute of Health, Berlin, Germany.

[6] German Center for Neurodegenerative Diseases (DZNE) Berlin, Berlin, Germany.

*Corresponding author. Email: carsten.finke@charite.de
[†]These authors contributed equally to this work.

Supplementary Text 1

A note on weighted permutation entropy and the occurrence of individual motifs

The mathematical formulation of weighted permutation entropy (WPE) relies on transforming the individual datapoints within a BOLD timeseries into rank space. We here employed a motif length of m = 3. In other words, three consecutive datapoints are considered and categorized based on their rank structure into one of 6 (m factorial) possible permutations. This categorization step (i.e., rank-encoding and amplitude weighting) is repeated along the timeseries, sliding by 1 datapoint and considering 3 datapoints each time, until the number of occurrences of each possible motif is counted. This results in a probability distribution that depicts the amplitude-weighted frequency of each pattern that occurred in the BOLD signal during a given timeframe. The Shannon entropy is then computed on this distribution, with the resulting value serving as the WPE estimation.

Within a given signal, this framework quantifies the amount of information encoded in the signal. We here apply a *time-resolved* calculation of WPE, using a sliding window method to segment each BOLD signal into overlapping windows. Thus, *within a given window*, a BOLD signal that is constantly increasing as it approaches an inflection point will have a high frequency of the [1, 2, 3] motif and low frequencies for every other pattern, whereas another BOLD signal that is constantly decreasing after an inflection point will have a high frequency of the [3, 2, 1] motif and low (or no) frequencies for every other pattern. Inflection points in the signal are characterized by the rarer motifs: [1, 3, 2], [2, 3, 1], [2, 1, 3], and [3, 1, 2] (Fadlallah et al., 2013; Krohn et al., 2023).

In this toy example, let the frequency distributions of these two signal snippets (monotonically increasing, monotonically decreasing) be exact mirror opposites of one another. Importantly, in the information theoretic sense, both exemplar signals are highly predictable and carry a low amount of information; in fact, their WPE values are close to zero and exactly the same. The WPE algorithm is not sensitive to the occurrence of individual motifs within a pattern frequency distribution, and thus quantifies the amount of information carried over the distribution of all possible patterns. On the contrary, the toy BOLD signals described above would necessarily have a low level of covariance and thus a FC estimate close to zero. This exemplar scenario presents a case in which the synchronization between a pair of BOLD signals is low, yet in the information theoretic sense, the signals are equally complex (or *not* complex). This toy example underscores the utility of a novel metric that captures information about the similarity of BOLD patterns and that which can be used to link underlying signal properties to both changes in nodal complexity and connectivity between signals. Our so-called 'index of pattern incongruency' (IPI) aims to address this gap.

Supplementary Methods 1

Noise simulations

To assess the robustness of IPI, AUC-SCD, and WPE to additive noise in the underlying BOLD signals, we conducted a systematic simulation of noise effects under various injection conditions in our cohort of 75 HCs. A set of 30 edges was randomly selected, and Gaussian white noise was added to the original, preprocessed BOLD signals. Injected noise was scaled by the signal's standard deviation and parametrized by a noise level alpha (α), ranging from 0 to 1 in increments of 0.1. Noise was injected under three complementary conditions: (1) noise added to the first region only, (2) noise added to the second region only, and (3) noise added to both regions simultaneously, and the complete complexity analysis pipeline was recomputed, including calculations of time-resolved WPE, AUC-SCD, and IPI.

These control analyses confirm that WPE estimates positively scale with increasing levels of noise in the underlying BOLD signals (Fig S3, panel A, left), which aligns with results from previous analyses in Krohn et al. (2023, Supplementary Material, Fig. S3). Overall, AUC-SCD and IPI measures were robust to low and moderate levels of noise, but tended to plateau around noise levels of $\alpha=0.5$ and $\alpha=0.7$, respectively (Fig S3., panel A, right). Methodological schematics of two exemplar BOLD timeseries under increasing additive noise schemes ($\alpha=0$, $\alpha=0.5$, $\alpha=1$), as well as their corresponding WPE timeseries, cofluctuations, and AUC-SCD profiles are additionally depicted in Fig. S3, panels B and C.

Supplementary Figures

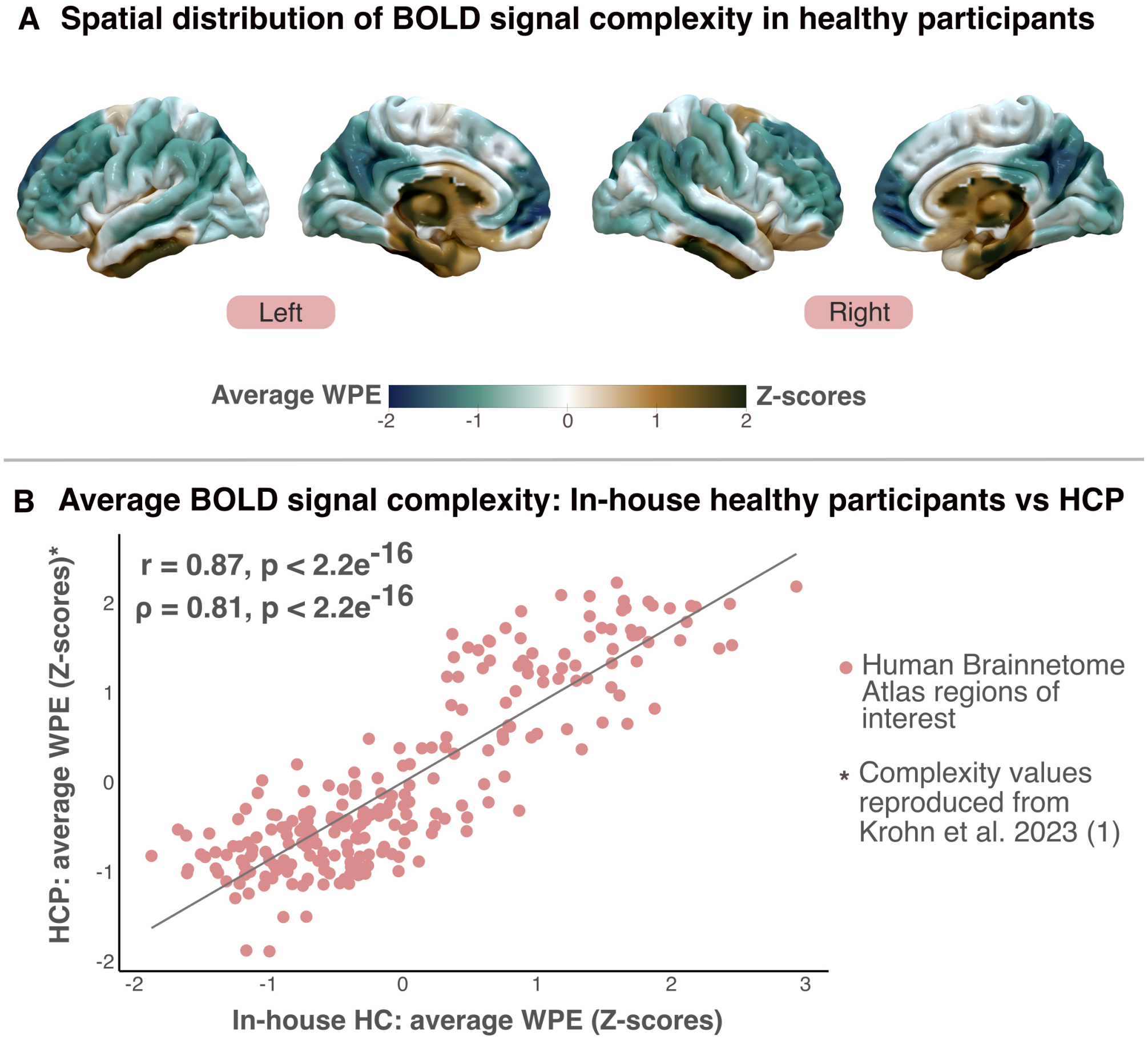

Fig. S1. Average signal complexity of in-house healthy participants is consistent with findings from the HCP Young Adult dataset. (A) Mean BOLD signal complexity values (WPE) computed over healthy control participants (n = 75) and mapped to brain space. Mean WPE values were Z-scored over regions. Gold colors represent high complexity and blue colors represent low complexity. **(B)** Correlation between the average complexity values displayed in (A) and the re-computed average complexity values from the Human Connectome Project (HCP) Young Adult dataset (Van Essen et al., 2012), originally analyzed in (Krohn et al., 2023). Both analyses applied the same parcellation schema using the Human Brainnetome Atlas (Fan et al., 2016), thus the spatial topologies of WPE values could be directly compared. Strong positive correlation coefficients indicate that the spatial distribution of WPE is highly consistent between datasets.

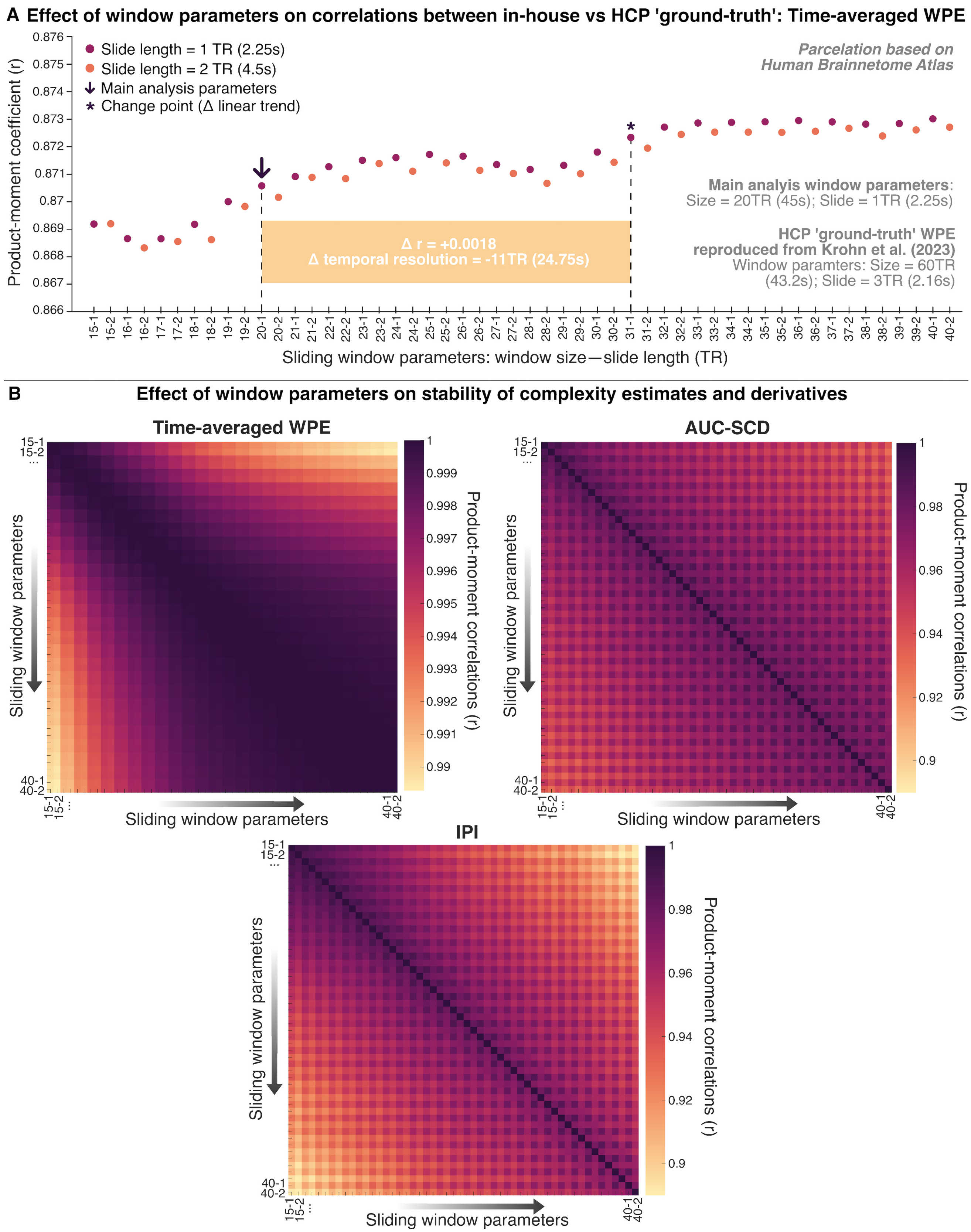

Fig. S2. Sensitivity of complexity estimates and derivatives to variations in BOLD signal sliding window parameters. Time-resolved WPE, AUC-SCD, and IPI were systematically assessed in a series

of sliding window parameter trials applied to the segmentation of underlying BOLD signals. Using window sizes from 15 TR (33.75s) to 40 TR (90s) and slide lengths of 1 TR (2.25s) and 2 TR (4.5s), BOLD data from 75 healthy participants was repeatedly segmented using a sliding window approach and underwent complexity analysis, as applied in the main text (see Materials and Methods for details). This produced a dataset of 52 trials that was used for subsequent sensitivity analyses. (A) Spatial consistency between in-house WPE estimates and 'ground-truth' WPE estimates (HCP Young Adult dataset, see Krohn et al. 2023) is robust to variations in BOLD signal windowing parameters. Scatter plot depicts trial-wise spatial correlations between in-house average WPE and 'ground-truth' WPE. The trial corresponding to the parameters used in the main analyses is indicated with a downward arrow (20-1), and the slope changepoint corresponding to a maximal spatial correlation is indicated with a star. (31-1). At this changepoint, we observe a minimal increase ($\Delta r = 0.0018$) in the spatial consistency of WPE values and a loss of temporal resolution of 11TR (24.75s), compared to the parameters used in our main analyses. (B) Spatial patterns of WPE, AUC-SCD, and IPI are highly robust to variations in sliding window parameters. Heatmaps depict pair-wise product-moment correlations between mean spatial maps between trials. For WPE, all correlations were above 0.98, and for AUC-SCD and IPI all correlations were above 0.92 and 0.88, respectively.

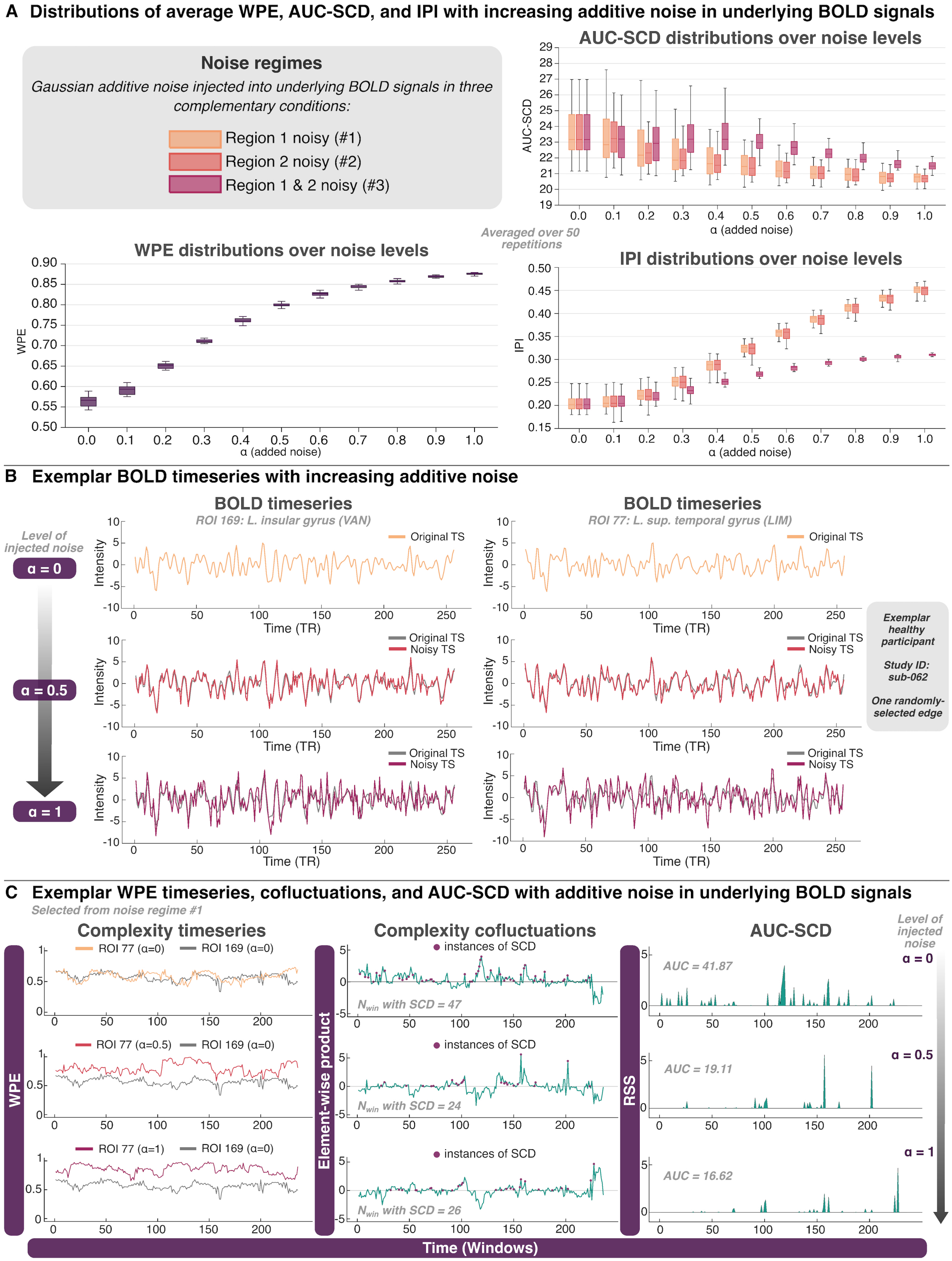

Fig. S3. Sensitivity of complexity estimates, cofluctuations, and pattern incongruency to noise injections in underlying BOLD signals. (A) Gaussian white noise was injected into preprocessed

BOLD signals from 30 randomly selected region pairs, scaled by the signal's standard deviation and parametrized by a noise level alpha (α) ranging from 0 to 1 in increments of 0.1. Noise was injected under three complementary conditions: (1) noise added to the first region only, (2) noise added to the second region only, and (3) noise added to both regions simultaneously. For each condition and noise level, 50 independent noise realizations were generated and subsequently averaged to produce stable regional distributions of WPE and edgewise distributions of AUC-SCD and IPI. (Left) WPE increased monotonically with noise strength, confirming that the noise manipulation systematically increased signal irregularity and providing a validation of the noise-injection procedure; this finding aligns with results from our previous analyses in Krohn et al. (2023, Supplementary Material, Fig. S3). (Right, top) AUC-SCD exhibited a gradual decrease with increasing noise strength across all conditions, consistent with the notion that additive noise increasingly disrupts the temporal alignment of complexity dynamics between regions, thereby reducing the magnitude and duration of simultaneous complexity decreases. (Right, bottom) IPI increased monotonically with noise strength, particularly under asymmetric noise conditions, which is consistent with its conceptualization as a measure of pattern incongruency. Under symmetric noise conditions, AUC-SCD and IPI measures were robust to low and moderate levels of noise, but tended to plateau around $\alpha=0.5$ and $\alpha=0.7$, respectively. **(B)** Exemplar BOLD timeseries from two brain regions, depicted (i) as used in main analyses in their original, preprocessed form ($\alpha=0$; top), (ii) with a moderate level of injected noise ($\alpha=0.5$; middle), and (iii) with a high level of injected noise ($\alpha=1.0$; bottom). **(C)** Exemplar complexity timeseries, cofluctuations, and AUC-SCD profiles corresponding to the pair of regions depicted in panel B and shown for increasing levels of injected noise ($\alpha=0$, top; $\alpha=0.5$, middle; $\alpha=1.0$, bottom). For visualization purposes, timeseries were selected from one iteration of trials under noise regime #1, where noise was injected asymmetrically.

Supplementary Tables

Table S1. Hippocampal functional connectivity alterations in patients with NMDAR encephalitis.

Seed region	Anatomical label	Detailed anatomical and modified cyto-architectonic description	Network label	MNI coordinates (X, Y, Z)	T	p_{uncor}	p_{FDR}	Effect size (Cohen's D)
L. HPC	L. sup. frontal gyrus	A9m, medial area 9	DMN	-5, 36, 38	3.12	<0.001	0.026	0.399
	L. sup. frontal gyrus	A10m, medial area 10	DMN	-8, 56, 15	3.74	<0.001	0.004	0.459
	R. sup. frontal gyrus	A10m, medial area 10	DMN	8, 58, 13	2.92	0.002	0.049	0.376
	L. orbital gyrus	A14m, medial area 14	DMN	-7, 54, -7	3.58	<0.001	0.014	0.451
	R. orbital gyrus	A14m, medial area 14	DMN	6, 47, -7	3.72	<0.001	0.004	0.503
	L. cingulate gyrus	A23d, dorsal area 23, posterior	DMN	-4, -39, 31	2.91	0.002	0.049	0.320
	L. cingulate gyrus	A32p, pregenual area 32, anterior	DMN	-6, 34, 21	4.25	<0.001	<0.001	0.531
	R. cingulate gyrus	A32p, pregenual area 32	VAN	5, 28, 27	2.88	0.002	0.049	0.392
	L. cingulate gyrus	A23v, ventral area 23	DMN	-8, -47, 10	3.09	0.002	0.043	0.396
	L. cingulate gyrus	A32sg, subgenual area 32, anterior	DMN	-4, 39, -2	4.27	<0.001	<0.001	0.529
	R. cingulate gyrus	A32sg, subgenual area 32, anterior	DMN	5, 41, 6	4.03	<0.001	0.003	0.491
	L. thalamus	mPFtha	THA	-7, -12, 5	4.16	<0.001	0.003	0.600
R. HPC	L. sup. frontal gyrus	A9m, medial area 9	DMN	-5, 36, 38	3.11	0.001	0.027	0.396
	R. sup. frontal gyrus	A9m, medial area 9	FPN	6, 38, 35	3.04	0.001	0.029	0.392
	L. sup. frontal gyrus	A10m, medial area 10	DMN	-8, 56, 15	3.03	0.001	0.029	0.357
	R. sup. frontal gyrus	A10m, medial area 10	DMN	8, 58, 13	2.76	0.003	0.042	0.347
	L. orbital gyrus	A14m, medial area 14	DMN	-7, 54, -7	3.24	0.001	0.027	0.402
	R. orbital gyrus	A14m, medial area 14	DMN	6, 47, -7	3.66	<0.001	0.007	0.498
	R. cingulate gyrus	A24rv, rostroventral area 24	FPN	5, 22, 12	2.87	0.002	0.033	0.432
	L. cingulate gyrus	A32p, pregenual area 32	DMN	-6, 34, 21	4.45	<0.001	<0.001	0.540
	R. cingulate gyrus	A32p, pregenual area 32	VAN	5, 28, 27	3.84	<0.001	0.002	0.493
	L. cingulate gyrus	A23v, ventral area 23	DMN	-8, -47, 10	3.22	<0.001	0.012	0.396
	R. cingulate gyrus	A23v, ventral area 23	VIS	9, -44, 11	2.82	0.002	0.033	0.381
	L. cingulate gyrus	A32sg, subgenual area 32	DMN	-4, 39, -2	4.23	<0.001	<0.001	0.538
	R. cingulate gyrus	A32sg, subgenual area 32	DMN	5, 41, 6	4.19	<0.001	<0.001	0.529
	L. thalamus	mPFtha	THA	-7, -12, 5	3.85	<0.001	0.002	0.567
	R. thalamus	mPFtha	THA	7, -11, 6	3.07	<0.001	0.033	0.454
	R. thalamus	rTtha, rostral temporal thalamus	THA	3, -13, 5	3.27	<0.001	0.019	0.509

NMDAR: anti-N-methyl-D-aspartate receptor; L: left hemisphere; R: right hemisphere; HPC: hippocampus; sup: superior; mPFtha: medial prefrontal thalamus; DMN: default mode network; VAN: ventral attention network; THA: thalamus; FPN: frontoparietal network; VIS: visual network.

Table S2. Hippocampal BOLD pattern incongruency (IPI) alterations in edges with reduced functional connectivity in NMDAR encephalitis

Seed region	Anatomical label	Detailed anatomical and modified cyto-architectonic description	Network label	MNI coordinates (X, Y, Z)	T	p_{uncor}	p_{FDR}	Effect size (Cohen's D)
L. HPC	L. sup. frontal gyrus	*A10m, medial area 10*	DMN	-8, 56, 15	-2.32	0.012	0.028	-0.357
	L. orbital gyrus	*A14m, medial area 14*	DMN	-7, 54, -7	-2.70	0.004	0.012	-0.378
	R. orbital gyrus	*A14m, medial area 14*	DMN	6, 47, -7	-2.98	0.002	0.012	-0.404
	L. cingulate gyrus	*A23d, dorsal area 23*	DMN	-4, -39, 31	-2.24	0.014	0.029	-0.331
	L. cingulate gyrus	*A32p, pregenual area 32*	DMN	-6, 34, 21	-2.07	0.021	0.033	-0.365
	L. cingulate gyrus	*A23v, ventral area 23*	DMN	-8, -47, 10	-2.79	0.003	0.012	-0.343
	L. cingulate gyrus	*A32sg, subgenual area 32*	DMN	-4, 39, -2	-2.09	0.022	0.033	-0.296
	L. thalamus	*mPFtha*	THA	-7, -12, 5	-2.74	0.003	0.012	-0.492
R. HPC	L. cingulate gyrus	*A32sg, subgenual area 32*	DMN	-4, 39, -2	-2.53	0.007	0.037	-0.372
	L. thalamus	*mPFtha*	THA	-7, -12, 5	-3.13	0.001	0.011	-0.569
	R. thalamus	*rTtha, rostral temporal thalamus*	THA	3, -13, 5	-3.16	0.001	0.011	-0.522

NMDAR: anti-N-methyl-D-aspartate receptor; L: left hemisphere; R: right hemisphere; HPC: hippocampus; sup: superior; mPFtha: medial prefrontal thalamus; DMN: default mode network; VAN: ventral attention network; THA: thalamus; FPN: frontoparietal network; VIS: visual network.

Supplementary References

Fadlallah, B., Chen, B., Keil, A., & Príncipe, J. (2013). Weighted-permutation entropy: A complexity measure for time series incorporating amplitude information. *Physical Review E*, *87*(2), 022911. https://doi.org/10.1103/PhysRevE.87.022911

Fan, L., Li, H., Zhuo, J., Zhang, Y., Wang, J., Chen, L., Yang, Z., Chu, C., Xie, S., Laird, A. R., Fox, P. T., Eickhoff, S. B., Yu, C., & Jiang, T. (2016). The Human Brainnetome Atlas: A New Brain Atlas Based on Connectional Architecture. *Cerebral Cortex (New York, NY)*, *26*(8), 3508–3526. https://doi.org/10.1093/cercor/bhw157

Krohn, S., von Schwanenflug, N., Waschke, L., Romanello, A., Gell, M., Garrett, D. D., & Finke, C. (2023). A spatiotemporal complexity architecture of human brain activity. *Science Advances*, *9*(5). https://doi.org/10.1126/sciadv.abq3851

Van Essen, D. C., Ugurbil, K., Auerbach, E., Barch, D., Behrens, T. E. J., Bucholz, R., Chang, A., Chen, L., Corbetta, M., Curtiss, S. W., Della Penna, S., Feinberg, D., Glasser, M. F., Harel, N., Heath, A. C., Larson-Prior, L., Marcus, D., Michalareas, G., Moeller, S., … Yacoub, E. (2012). The Human Connectome Project: A data acquisition perspective. *NeuroImage*, *62*(4), 2222–2231. https://doi.org/10.1016/j.neuroimage.2012.02.018
